# Innate Immunity Communicates Using the Language of Extracellular Microvesicles

**DOI:** 10.1007/s12015-021-10138-6

**Published:** 2021-02-25

**Authors:** Mariusz Z. Ratajczak, Janina Ratajczak

**Affiliations:** 1 Stem Cell Institute at James Graham Brown Cancer Center, University of Louisville, 500 S. Floyd Street, Rm. 107, Louisville, KY 40202 USA; 2 Department of Regenerative Medicine, Center for Preclinical Research and Technology, Medical University of Warsaw, Warszawa, Poland

**Keywords:** ExMVs, Exosomes, Innate immunity, Complement cascade, RNA, Horizontal transfer of RNA

## Abstract

The innate immunity system and extracellular microvesicles (ExMVs) both emerged early in the evolution of life, which is why its innate immunity cellular arm and its soluble-component arm learned, understood, and adapted to the “language” of ExMVs. This was most likely the first language of cell–cell communication during evolution, which existed before more specific intercellular crosstalk involving specific ligands and receptors emerged. ExMVs are involved in several processes in the body, including immune and coagulation responses, which are part of inflammation. In this review we will briefly highlight what is known about how ExMVs regulate the function of the cellular arm of innate immunity, including macrophages, monocytes, granulocytes, natural killer cells, and dendritic cells, and affect the soluble components of this system, which consists of the complement cascade (ComC) and soluble, circulating, pattern-recognition receptors (collectins, ficolins, and pentaxrins). These effects are direct, due to the fact that ExMVs affect the biological functions of innate immunity cells and may directly interact with soluble components of this system. Moreover, by activating coagulation proteases, ExMVs may also indirectly activate the ComC. In this review, we will use the term “extracellular microvesicles” (ExMVs) to refer to these small, spheroidal blebs of different sizes, which are surrounded by a membrane lipid layer. We will focus on the role of both ExMVs released during cell-surface membrane budding and smaller ExMVs, known as exosomes, which are derived from the budding of the endosomal membrane compartment. Finally, we will provide a brief update on the potential therapeutic applications of ExMVs, with a special emphasis on innate immunity.

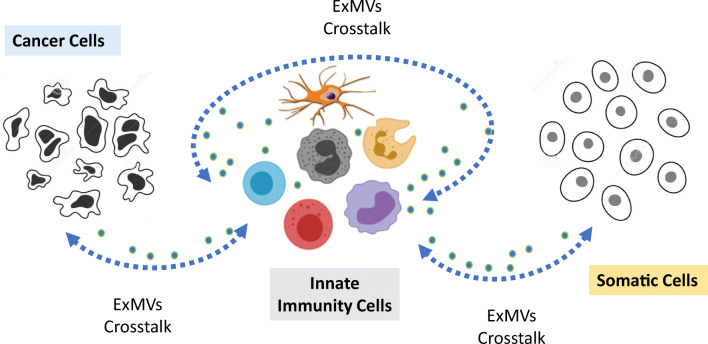

## Introduction

The pleiotropic responses of the immune system are regulated, on the one hand, by inborn or innate immunity and, on the other hand, by acquired or adaptive immunity [1, 2]. Inborn or innate immunity, which is the topic of the current review, consists of cellular and humoral arms. The cellular arm consists of several cell types, including neutrophils, monocytes, basophils, and eosinophils as well as mast, natural killer, dendritic, and gamma/delta T cells [1, 2]. These cells respond to changes in the microenvironment caused by infectious pathogens, circulating soluble mediators, physical and metabolic stimuli, and, what is the topic of this review, the release of extracellular microvesicles (ExMVs), which are small, bilayer membrane-covered blebs circulating in biological fluids [3–16]. The innate immunity cells of the cellular arm secrete ExMVs in response to external stimuli and, along with soluble mediators, are major components of the secretome [17]. By contrast, the humoral arm of innate immunity consists of protein components, and the complement cascade (ComC) is its most prominent part. The ComC becomes activated in the (i) classical, (ii) mannan-binding lectin, and (iii) alternative pathways [1, 2]. Activation of these pathways leads to cleavage of the C3 and C5 components of the ComC; release of the C3a, _desArg_C3a, C5a, and _desArg_C5a anaphylatoxins; and formation of C5b-C9, also known as the membrane attack complex (MAC). In addition to the ComC, the humoral arm of innate immunity also consists of soluble receptors, such as collectins, ficolins, and pentraxins, that circulate in peripheral blood (PB) and recognize danger-associated molecular pattern molecules (DAMPs) or alarmins[1, 2]. While pentraxins behave as the functional ancestors of antibodies (Abs), collectins and, in particular, one of the ficolins known as mannan-binding lectin (MBL) play an important role in triggering the mannan-binding lectin pathway of ComC activation [1, 2]. It is well established that ExMVs secreted by innate immunity cells are involved in cell–cell communication during coordinated immune responses. ExMVs also directly or indirectly modulate activities of components of the soluble arm of innate immunity [3–16]. Furthermore, they may also affect responses of the acquired adaptive immune system represented by B and T lymphocytes, contributing to regulatory crosstalk between inborn and acquired immunity [3–17].

ExMVs have become of great interest to basic researchers and clinicians as important players in maintaining tissue homeostasis, cell differentiation, as well as organ development and remodeling. They are also involved in pleiotropic inflammatory responses. These small, spheroidal, membrane-coated vesicles are detectable under steady-state conditions in all the biological fluids investigated so far, including blood plasma, intercellular fluid, lymph, cerebrospinal fluid, bile, synovial fluid, saliva, urine, sperm, and breast milk [17–21]. Their level is elevated in PB and other biological fluids in response to infections, autoinflammatory diseases, tissue or organ injuries, and malignancies [21–29]. This increase occurs in parallel with activation of innate immunity responses, leading to activation of the ComC and the coagulation cascade (CoaC). These observations and correlations between levels of circulating ExMVs and activation of the ComC and CoaC support an important modulatory role for ExMVs in orchestrating immune responses [3–16].

It is known that ExMVs come together in biological fluids as a mixture of large and small vesicles. It is difficult to separate them, and it must be stressed that the real biological impacts of these variable-sized vesicles on biological processes must be considered together, even if there are obvious differences in their size and molecular compositions [17–22]. The size of ExMVs depends on their origin in a given cell compartment. For example, the larger ExMVs are released during cell-surface membrane budding. The size of these ExMVs is in the range of 100–1000 nm in diameter, and they are composed of an outer lipid bilayer, which surrounds the inner content composed of different molecules, including various mRNA species (e.g., coding RNA, miRNA, noncoding RNA, and circular RNA), proteins (e.g., enzymes, signaling mediators, and transcription factors), bioactive lipids (e.g., sphingosine-1-phosphate, prostaglandins, and leukotrienes), signaling nucleotides (extracellular ATP and extracellular adenosine), and metabolites [17–38]. Large ExMVs may even contain organelles (e.g., mitochondria) hijacked from the cell cytoplasm during their formation. By contrast, the smaller ExMVs, or exosomes, are derived from the endosomal cell membrane compartment. They are generated by budding of the endosomal membranes toward the interior of the endosome, which creates endosomal multi-vesicular bodies (MvBs) enriched in small (~ 50–150 nm in diameter) intraluminal vesicles that are the precursors of exosomes. After fusion with the cell-surface plasma membrane these MVBs release their content of small intraluminal exosomes into the extracellular space [30–38].

Importantly, both large and small ExMVs circulating in biological fluids co-regulate the biological responses mediated by innate immunity. They also affect the function of the acquired immune system, although this will not be discussed in this review.

### Innate Immunity Cells Communicate with Each Other by Means of ExMVs, Respond to ExMVs Released by Other Cells, and Send ExMV-based Messages in Return

**Fig. 1:**
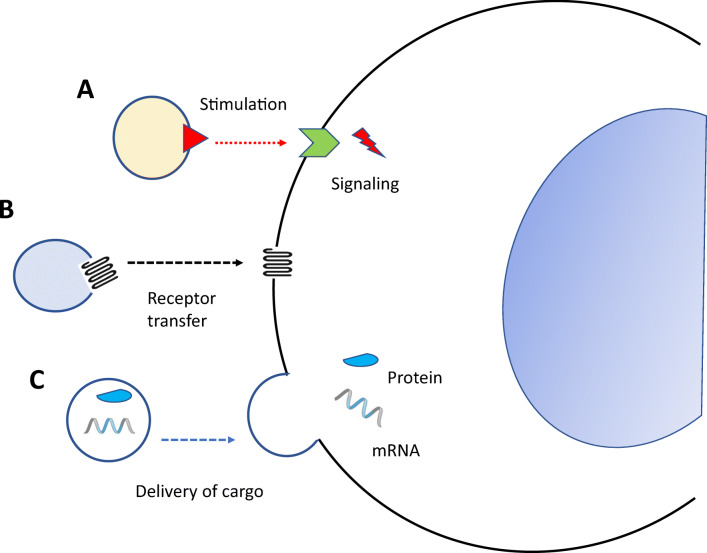
Biological effects of ExMVs on target cells. ExMVs may interact with receptors expressed on target cells by means of surface-expressed ligands (**a**), transfer receptors to the target cell plasma membrane (**b**), or transfer cargo containing mRNA, miRNA, proteins, or other biomolecules from one cell to another (**c**)

ExMVs most likely served as the first language with which cells started to communicate with each other before more specific mechanisms involving various classes of ligands, including peptide-based cytokines, chemokines, growth factors, steroid hormones, bioactive lipids, extracellular nucleotides, and their specific receptors, emerged during evolution [17, 27]. The biological effects of ExMVs are characterized by their pleotropic roles in cell–cell communication (Fig. 1). First, they serve as signaling platforms, in which they stimulate cells with ligands embedded in their outer lipid layer. Various peptide-based and non-peptide-based ligands have been identified on their surfaces (Fig. 1a). Second, they may also act as cell-surface phenotype “modifiers”, by transferring cell membrane receptors between cells. An example is the transfer of certain chemokine receptors or adhesion molecules between cells by means of ExMVs (Fig. 1b). Finally, ExMVs can be considered as cargo-delivery packets, by delivering to the target cells their content of mRNA species, proteins, bioactive lipids, and signaling nucleotides. By employing this mechanism, ExMVs may modify the biochemistry and epigenetics of target cells (Fig. 1c). Depending on their size, the cargo transferred by ExMVs becomes internalized after delivery into the cells by various mechanisms, including phagocytosis; caveolin-, clathrin-, or lipid raft-mediated endocytosis; micropinocytosis; and direct membrane fusion. If ExMVs are not degraded in the cell lysosomal compartment, they release their intact biologically active cargo into the cytosol of the target cells. This way, they play a role in the horizontal transfer of several bioactive molecules from one cell to another [30–38].

**Fig. 2:**
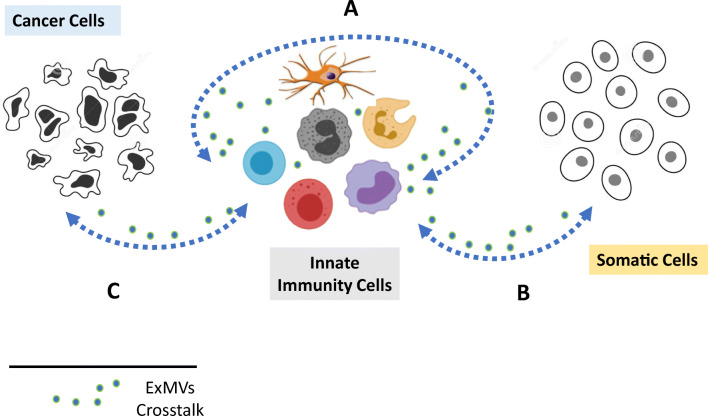
The role of ExMVs in innate immunity cell–cell communication.**a** Innate immunity cell-secreted ExMVs are involved in mutual cell**–**cell communication between these cells. **b** Using ExMVs, innate immunity cells exchange information with other somatic cells. These interactions involve mutual exchange of ExMV-based messages. **c** Innate immunity cells have crosstalk with cancer cells by means of ExMVs. Again, this crosstalk follows the principle of a “two-way street”

It is well known that cells belonging to the cellular arm of innate immunity release ExMVs if the cells are exposed to pathogens, pathogen-associated molecular patterns (PAMPs), or other stressors, such as danger-associated molecular patterns (DAMPs) or alarmins. As shown in Fig. 2, innate immunity cells (i) communicate with each other by exchanging ExMVs, (ii) respond to ExMVs secreted from somatic cells (e.g., mesenchymal stromal cells or cancer cells), and (iii) send ExMV-based messages to these cells in return [3–16]. This communication is intense whenever there is activation of the immune system, as seen in pathogen infection, sterile inflammation, organ or tissue injuries, and tumor growth. We envision that the intercellular environment becomes enriched in circulating ExMVs of different sizes in all these processes. Depending on their cell of origin and molecular composition, ExMVs are responsible for pleiotropic biological effects, and it is still somewhat difficult to fully decipher the meaning of all of these interactions. In order to isolate causes and effects, most of the available experimental results are obtained from controlled in vitro experiments, often employing purified fractions of ExMVs. What is important to keep in mind, ExMVs are only part of the entire secretome, which also includes soluble molecules, and final biological effects are the response to both secretome components.

The currently available approaches to characterizing ExMVs include (i) western blot to detect teraspannin components, in the case of exosomes, or certain cytoskeletal proteins expressed in cell membrane-derived ExMVs, (ii) nanoparticle tracking analysis, to calculate size distributions and numbers of ExMVs in suspension, and (iii) electron microscopy-based approaches and flow cytometry combined with antibodies against surface markers, suitable for characterization of larger ExMVs [17–22, 34]. There are also strategies to analyze the molecular signatures of ExMVs based on analysis of their cargo by employing “omics” technologies aimed at the universal detection of mRNA species (transcriptomics), proteins (proteomics), lipids (lipidomics), and metabolites (metabolomics). The evidence for the biological effects of ExMVs in innate immunity is expanding rapidly, and, due to space limitations, we will highlight just a few recent representative observations of the biological effects of innate immunity cells releasing ExMVs.

#### Neutrophil-derived ExMVs

Of all the innate immunity cells, neutrophils are the most important source of ExMVs [8–10, 15]. They produce these spheroidal membrane structures in response to various stimuli, including pathogens, cytokines, chemokines, ComC cleavage fragments, and antibodies. The ExMVs derived from these cells have been demonstrated to bind specifically to other innate immunity cells, such as macrophages/monocytes and dendritic cells, and thereby alter their biology and function. As recently proposed, they can be categorized into two subtypes according to the mechanism by which they are generated and the biological role they fulfill, namely, as (i) neutrophil-derived microvesicles and (ii) neutrophil-derived trails [15]. They have different biological functions, as the first type of neutrophil-derived ExMVs is found in tissues to which these cells migrate and are often anti-inflammatory, whereas the second type of ExMVs is endowed with pro-inflammatory properties and is found in the inflammatory areas where neutrophils had previously arrived to fulfill their tasks.

To explain these two types of ExMVs and their opposite effects, successful inflammation requires effective initiation and subsequent resolution of the inflammatory process. Neutrophils are the first cells of the innate immunity cellular arm recruited to sites of injury or infection and initiate inflammation to eliminate pathogens, release chemokines and cytokines, and, by secreting ExMVs, modulate the functions of other cells involved in the immune reponse. Subsequently, they are also involved in the resolution of inflammation by releasing anti-inflammatory ExMVs—described as neutrophil-derived trails, as mentioned above [15]. At the molecular level, these different types of neutrophil-derived ExMVs are enriched in various types of miRNAs that, after transfer to macrophages, affect macrophage phenotypic polarization toward either a proinflammatory phenotype or an anti-inflammatory phenotype [8–10]. However, despite the fact that these two types of ExMVs, play different roles in modulating immune responses, they still have bactericidal activity due to the release of reactive oxygen species (ROS) and granule-secreted myeloperoxidase, lactoferrin, elastase, and proteinase activities. Moreover, they also chemoattract monocytes. Neutrophil-derived ExMVs also express phosphatidyl serine, activate the CoaC, and thereby promote coagulation. As in the case of many cell types, their release is stimulated by the C5a cleavage fragment released by the activated ComC. Furthermore, recent clinical results revealed that neutrophil-derived ExMVs activate monocytes and drive them into a pro-inflammatory phenotype, which, unfortunately, is associated with a higher mortality rate in intensive care unit patients [8]. Therefore, more work is needed to better understand the role of this particular type of ExMVs in human pathologies.

#### Monocyte/macrophage-derived ExMVs

Another type of innate immunity cells that are a rich source of ExMVs are macrophages [11–13]. This is a highly heterogenous cell population derived from the precursors of the myeloid lineage, with the capability of differentiating into two activated macrophage subtypes, M1-like and M2-like. M1 macrophages are associated with Th1 responses and secrete large amounts of pro-inflammatory factors, such as tumor necrosis factor alpha (TNF-α) and interleukin 1 beta (IL-1β), in addition to ROS. By contrast, M2 macrophages are anti-inflammatory cells producing interleukin 10 (IL-10) and are involved in tissue tolerance, repair, and remodeling. Proteomic studies on both monocyte- and macrophage-derived ExMVs revealed that they contain a large variety of DAMPs or alarmins, including galectins, annexins, heat-shock proteins, S100-family proteins, cathelicidin, defensin, α3 endoplasmin, and high molecular group box 1 protein (HMGB1). Since alarmins can bind a range of receptors, including Toll-like receptors (TLRs) and receptors for advanced glycosylation end products (RAGE), they play a robust role in initiating crosstalk between innate immunity cells [13, 14]. Based on the fact that macrophages can be roughly divided into the two abovementioned subtypes, M1-like and M2-like, ExMVs derived from these different types of macrophages carry different biological cargos and mediate different biological effects. Since macrophage-derived ExMVs deliver mRNA species, proteins, bioactive lipids, and signaling nucleotides to modify the phenotype and function of target cells, their cargo may vary with different phenotypes (M1-like or M2-like) or with the microenvironments in which they originate. Therefore, it is understandable that ExMVs derived from polarized or naïve macrophages display distinct regulatory miRNA profiles. Taking into consideration that macrophages usually present mixed phenotypes in response to different diseases or different phases of disorders, it is not easy to separately identify the contents of M1 or M2 monocyte-derived ExMVs. Another question is related to activation of an intracellular complex in macrophages known as the Nlrp3 inflammasome [1, 2]. This may lead to pyroptosis of these cells and the release of several types of DAMPs from these cells as well as activated oligomeric Nlrp3 inflammasome complexes known as “specks”. After internalization by surrounding macrophages, specks may amplify the inflammatory response, and this phenomenon most likely occurs by ExMV-mediated transfer of Nlrp3 inflammasome specks [39].

#### Dendritic Cell-derived ExMVs

Another important cellular component of the cellular arm of innate immunity is dendritic cells [6, 7]. These play a central role in initiating and regulating immune responses against cancer cells. The ExMVs generated by dendritic cells express major histocompatibility complex (HLA) peptides and co-stimulatory molecules on their surface, which promotes their interaction with other immune cells, including CD8^+^ T cells and natural killer (NK) cells, and these are involved in rejecting growing malignancies. This latter phenomenon is explained by the fact that dendritic cell-derived ExMVs express ligands for NK cells on their surface, and thus they promote stimulation of these cells for antitumor responses. As discussed later in this review, these properties of dendritic cell-derived ExMVs make them interesting candidates for the development of new cancer treatment approaches, such as cancer vaccines and immunotherapeutics [6, 7, 11]. Interestingly, cancer cells may oppose this beneficial anti-tumor effect of dendritic cell-derived ExMVs by secreting tumor-derived ExMVs bearing a variety of molecules that block their function and may even promote the generation of myeloid-derived suppressor cells. Moreover, specific molecules expressed by tumor cell-derived ExMVs may be recognized by Toll-like receptors (TLRs) expressed on the surface of dendritic cells to trigger a signaling cascade that ultimately leads to tumor metastasis and downregulation of cytokine production [1, 2]. These ExMVs can also negatively modulate the immune response, for example, by sequestering antitumor antibodies on their surface and thus acting as decoys preventing these antibodies from coating malignant cells.

Finally, as shown in Fig. 2, the function of innate immunity cells may be affected by ExMVs secreted from other somatic cells. The best example is mesenchymal stromal cells (MSCs), which have anti-inflammatory properties. It is well known that several biological effects of MSCs as intact cells can be replaced by their ExMVs. Therefore, the biological immunomodulatory properties of MSCs can be conveyed by ExMVs derived from these cells. Examples are the role of MSC-derived ExMVs in attenuating graft-versus-host disease (GvHD) after bone marrow transplantation [29] and their potential role in inhibiting immune-mediated cytokine storms in patients during COVID-19 infection [40]. Overall, MSC-derived ExMVs may promote an immunosuppressive response through (i) induction of immature dendritic cells, (ii) polarization of macrophages toward an M2-like immunosuppressive phenotype, (iii) inhibition of immunoglobulin release, and (iv) promoting secretion of anti-inflammatory cytokines. In addition, recent findings indicate an impact of several miRNA species involved in MSC-derived ExMV-mediated anti-inflammatory effects on macrophages by regulating the M1/M2 balance. For example, it has been shown, that MSC-derived miR223 present as cargo in ExMVs may reprogram macrophages from the M1 to the M2 phenotype. Other candidate miRNAs mediating anti-inflammatory effects of these MSC-secreted ExMVs are miR-146b, miR-126, and miR-199a [3–16]. It is expected that this list is not complete and that new regulatory miRNA species will surely be identified.

In addition to macrophages, MSC-derived ExMVs also exert immunosuppressive effects on dendritic cells, primarily by inhibiting their activation, which prevents them from triggering T-cell-mediated responses. As reported, MSC-derived ExMVs impair (i) proliferation, (ii) cytotoxic degranulation, and (iii) IL-2-induced activation of both CD56-dim and CD56-bright NK cells, all in a TGF-b-dependent manner. In addition, these cells can also be inhibited in an IL-10- and HLA-G-dependent manner by the anti-inflammatory cargo of MSC-derived ExMVs [6].

#### The Complement Cascade (ComC) is Activated by ExMVs, and Active ComC Cleavage Fragments Promote the Release of ExMVs From Somatic Cells

**Fig. 3:**
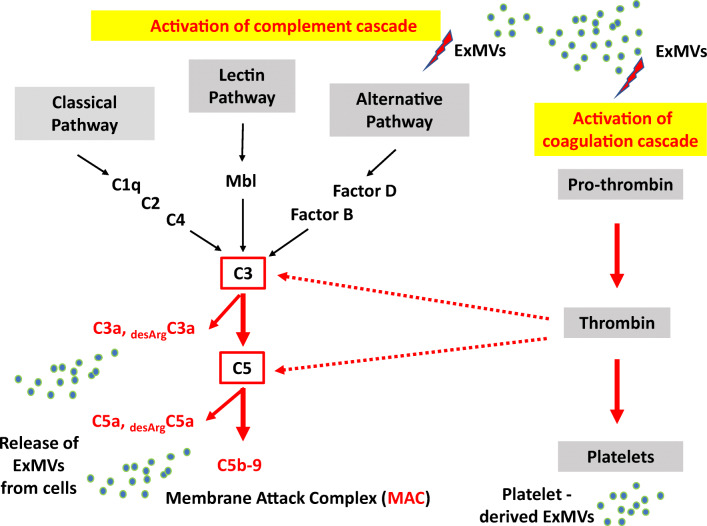
Complement and coagulation cascade crosstalk. Left panel. The ComC is activated by three pathways: the classical, mannan-binding lectin, and alternative pathways. Evidence has accumulated that ExMVs activate ComC activation. Moreover, ComC cleavage activates fragments, such as C3a, _desArg_C3a, C5, _desArg_C5a, and C5b-C9 (MAC), that activate immunity cells and somatic cells to release more ExMVs. Right panel. ExMVs also activate the coagulation cascade (CoaC). Thrombin generated during coagulation has intrinsic C3 and C5 convertase-like activity, contributing to activation of the ComC. Finally, thrombin activates blood platelets to release ExMVs

The ComC is an important part of the humoral arm of innate immunity as the initial barrier against pathogens [1, 2, 11]. As mentioned above, this cascade is activated by three different pathways, known as the classical, mannan-binding lectin, and alternative pathways (Fig. 3). These three pathways lead to the generation of C3 convertase, which subsequently cleaves the third protein component of the cascade (C3), a centrally important molecule, which after cleavage releases anaphylatoxins C3a and _desArg_C3a. In the following proteolytic reactions, the downstream part of the complement cascade becomes activated and forms C5 convertase, which cleaves the fifth protein component of complement (C5) and releases the other active anaphylatoxins, C5a and _desArg_C5a. Finally, after cleavage of C5, the terminal part of the ComC becomes activated, resulting in the formation of C5b-C9, also known as the membrane attack complex (MAC), which may lead to cell lysis. As shown in Fig. 3, both the C3 and C5 components of the ComC can also be cleaved by thrombin, which is a product of the activated CoaC and has C3a and C5a convertase-like activity [11]. This crosstalk supports the existence of an evolutionarily ancient functional connection between these proteolytic cascades.

What is important for the topic of this review, both cascades can be activated by ExMVs, as depicted in Fig. 3. This is not surprising, as several studies have reported increased numbers of circulating ExMVs in both inflammatory and thrombotic diseases. Nevertheless, the mechanisms of activation of the two cascades are different. Specifically, ExMVs trigger the classical pathway of ComC activation due to binding of C1q protein, which is the first component of this pathway, followed by activating elements further downstream in the cascade. Interestingly, the binding of C1q to ExMV membranes occurs through electrostatic interactions, rather than in an antibody-dependent manner. It has also been demonstrated that ExMVs also bind immunoglobulins (IgG) or C-reactive protein (CRP), which also triggers activation of the classical pathway of the ComC in a C1q-dependent manner [11].

In contrast to the ComC, ExMVs initiate activation of the CoaC and coagulation due to the fact that they express on their surfaces phosphatidyl serine (PS) and tissue factor (TF) [41, 42]. Both PS and TF serve as platforms for activation of the CoaC, which generates thrombin, a protease endowed with C3 and C5 convertase-like activity, as mentioned above (Fig. 3).

Moreover, since ComC activation leads finally to generation of its terminal product, which is cell-lytic MAC, the mechanism based on ExMV shedding in response to C3a and C5a allows MAC clearance from the plasma membrane, assuring cell survival and recovery from massive complement attack [11, 43, 44]. Interestingly, this C3a- and C5a-induced ExMV shedding, which is a common preventive measure to protect the cell from the MAC, was reported to occur in erythrocytes, granulocytes, glomerular epithelial cells, and in several established tumor cell lines. In addition to this ExMV-shedding mechanism, ExMVs may express key ComC factors and regulatory proteins on their surface, affecting ComC-mediated inflammation. By attracting and fixing ComC molecules and confining MAC formation to their surface, ExMVs may protect cells and tissues from MAC lysis [3–5, 11, 44].

Furthermore, if ExMVs express the complement regulatory molecules CD55 and CD59 on their surface, they may also modulate activation of the ComC. CD55 regulates the C3 and C5 convertases, whereas CD59 inhibits the formation of MAC. These findings strongly indicate that exosomes equipped with complement regulators, such as CD55 and CD59, are not only able to escape complement, but if they transfer these receptors after fusion with target cells, they may also protect these cells from MAC attack [43, 44].

What is also important, while ExMVs trigger activation of both the ComC and CoaC, active products of both cascades promote ExMV release from innate immunity and somatic cells [4]. These mutual interactions add to the complexity of ComC–ExMV interactions.

## Therapeutic Applications of Innate Immunity Cell-derived ExMVs

Based on the fact that ExMVs derived from immune cells, and specifically those derived from dendritic cells, appear to coordinate immune responses, these particular ExMVs have found therapeutic applications in cancer and auto-immune disorders as well as in vaccines for infectious diseases. For example, it has been demonstrated that dendritic cell-derived ExMVs loaded with tumor peptides promote rapid rejection of tumors in mice and have several advantages over cancer vaccines traditionally based on the application of intact dendritic cells [6]. This advantage is based on the fact that the molecular composition of ExMVs is more controllable than that of whole cells. Moreover, dendritic cell-derived ExMVs have a longer shelf life and a 10–100 times greater number of MHC complexes (involved in presenting tumor antigens to NK cells) per unit area than intact dendritic cells [6]. What is also important, these ExMVs are highly enriched in NK cell activation ligands.

Another therapeutic application of ExMVs could take advantage of the fact that that these small spheroidal vesicles are enriched in binding sites for complement molecules and could be employed as scavengers for soluble complement factors to protect patients from uncontrolled complement cascade activation, as seen for example in massive inflammation, sepsis, or cytokine storms [11]. The efficacy of this approach was established in in vitro experiments but needs further verification in well-controlled experiments in in vivo models.

Another important question is whether, in the case of the unwanted effects of innate immunity cell-derived ExMVs, it is feasible to remove them from circulation. In particular, it would be important to try this approach if they are involved in fatal complications during severe infections or tumor progression. To achieve this goal, there are, on the one hand, available potential strategies, including plasmapheresis and filtration, to decrease the burden of circulating ExMVs. On the other hand, other experimental treatments have been proposed that inhibit ExMV formation by therapeutic application of dimethyl amiloride or inhibit their fusion with target cells after the binding of ExMV-expressed phosphatidylserine with diannexin [17, 34]. This, however, requires further study before it can be proposed as a safe and efficient treatment in the clinic.

## Conclusions

Evidence has accumulated that ExMVs participate in almost all biological processes in the body, including immune and coagulatory responses. These important effects of ExMVs were unappreciated until recently. As discussed in this review, these remarkable structures regulate the function of the cellular components of innate immunity, including macrophages, monocytes, granulocytes, NK cells, and dendritic cells as well as soluble components of the innate immunity system, including the ComC. Nevertheless, further research is needed to better decipher the molecular signature of ExMV cargo derived from innate immunity cells, which includes RNA species (mRNA, miRNA, and long noncoding RNA), proteins, bioactive lipids, and signaling nucleotides. It is important to compare the molecular signatures of ExMVs isolated from normal human individuals with those from patients presenting various health problems. This knowledge could shed more light on the pathogenesis of various diseases and help to develop better therapeutic interventions. Despite some progress in the field, there are still many problems to be solved, including a lack of well-established, rapid, and standardized methods for isolating ExMVs; counting them; and purifying them efficiently from biological fluids. Thinking about the therapeutic application of innate immunity cell-derived ExMVs in the clinic, we also have to consider potential “off-target” side effects, including the risk of hypercoagulation. Nevertheless, there is no doubt that in the coming years we will witness the rapid development of ExMV-based therapeutic strategies and their diagnostic applications in the clinic, including in the exciting field of innate immunity.
